# Histological markers, sickle-shaped blood vessels, myxoid area, and infiltrating growth pattern help stratify the prognosis of patients with myxofibrosarcoma/undifferentiated sarcoma

**DOI:** 10.1038/s41598-023-34026-w

**Published:** 2023-04-25

**Authors:** Kota Washimi, Rika Kasajima, Eigo Shimizu, Shinya Sato, Yoichiro Okubo, Emi Yoshioka, Hiroto Narimatsu, Toru Hiruma, Kotoe Katayama, Rui Yamaguchi, Kiyoshi Yamaguchi, Yoichi Furukawa, Satoru Miyano, Seiya Imoto, Tomoyuki Yokose, Yohei Miyagi

**Affiliations:** 1 Department of Pathology, Kanagawa Cancer Center, Yokohama, Kanagawa Japan; 2 Molecular Pathology and Genetics Division, Kanagawa Cancer Center Research Institute, Yokohama, Kanagawa Japan; 3 Division of Health Medical Intelligence, Human Genome Center, Institute of Medical Science, The University of Tokyo, Tokyo, Japan; 4 Cancer Prevention and Control Division, Kanagawa Cancer Center Research Institute, Yokohama, Kanagawa Japan; 5 Division of Musculoskeletal Tumor Surgery, Kanagawa Cancer Center, Yokohama, Kanagawa Japan; 6 Division of Cancer Systems Biology, Aichi Cancer Center Research Institute, Nagoya, Japan; 7 Division of Cancer Informatics, Nagoya University Graduate School of Medicine, Nagoya, Japan; 8 Division of Clinical Genome Research, Institute of Medical Science, The University of Tokyo, Tokyo, Japan; 9 Department of Integrated Data Science, Medical and Dental Data Science Center, Tokyo Medical and Dental University, Tokyo, Japan

**Keywords:** Cancer, Risk factors

## Abstract

Myxofibrosarcoma (MFS) and undifferentiated sarcoma (US) have been considered as tumors of the same lineage based on genetic/epigenetic profiling. Although MFS shows a notably better prognosis than US, there are no clear criteria for distinguishing between them. Here, we examined 85 patients with MFS/US and found that tumors with infiltrative growth patterns tended to have more myxoid areas and higher local recurrence rates but fewer distant metastases and better overall survival. Morphologically characteristic sickle-shaped blood vessels, which tended to have fewer αSMA-positive cells, were also observed in these tumors, compared with normal vessels. Based on the incidence of these sickle-shaped blood vessels, we subdivided conventionally diagnosed US into two groups. This stratification was significantly correlated with metastasis and prognosis. RNA sequencing of 24 tumors (9 MFS and 15 US tumors) demonstrated that the proteasome, NF-kB, and VEGF pathways were differentially regulated among these tumors. Expression levels of *KDR* and *NFATC4*, which encode a transcription factor responsible for the neuritin-insulin receptor angiogenic signaling, were elevated in the sickle-shaped blood vessel-rich US tumors. These findings indicate that further analyses may help elucidate the malignant potential of MFS/US tumors as well as the development of therapeutic strategies for such tumors.

## Introduction

Myxofibrosarcoma (MFS) and undifferentiated sarcoma (US), formerly malignant fibrous histiocytoma (MFH), are among the most common adult soft tissue sarcomas^1^. Histologically, MFS is characterized by a myxoid stroma and infiltration into surrounding tissues^2^. Further, US, based on its current definition, may also include a myxoid stroma with high-grade sarcoma cells that lack a specific lineage^2^. MFH with myxoid stroma is considered MFS, with a better prognosis compared with other soft tissue sarcomas^3^. However, there are no clear criteria for distinguishing between US and MFS. A recent study based on The Cancer Genome Atlas (TCGA) datasets detailing the genetic/epigenetic profiles of undifferentiated pleomorphic sarcoma (UPS), a subtype of US and MFS, indicated that UPS is not a cancer type with distinct tumors and falls within a single spectrum of the disease^4^. Further, the World Health Organization (WHO) classification system does not clearly provide a cut-off for the proportion of myxoid stroma for discriminating MFS from US^2^.

The prognosis of MFS/US is variable; however, some cases without distant metastasis but with repeated local recurrence have a relatively good prognosis. Although distant metastasis indicates a poor prognosis in MFS/US^2^, the pathogenesis of distant metastases remains unknown. To address these issues, in the present study, we aimed to subclassify MFS/US based on histological features and correlate them with gene expression data to provide prognostic and systematic concepts regarding the disease.

## Methods

### Subjects

We reviewed archived pathology reports from the Kanagawa Cancer Center corresponding to the 2000–2018 period and identified cases that were diagnosed as MFS, UPS, US, or MFH. A single board-certified pathologists (K.W.), specializing in soft tissue tumor diagnosis, re-evaluated the pathology diagnoses and identified 85 MFS/US cases. We excluded cases of undifferentiated small round cell sarcoma. This study was approved by the institutional review board of Kanagawa Cancer Center (Approval number 2018 epidemiology-137), and we opened the information of this study and allowed the patients involved to opt-out from this study according to the instruction of the board.

### Histopathology

Pathological parameters were re-evaluated using hematoxylin–eosin (H&E)-stained samples of the largest cross-section of the tumor. Of all maximal sections, four did not show histological findings. Thus, for these cases, the histology of all the tumor area specimens (average of 12.75 blocks) was rechecked. The French Federation of Cancer Centers Sarcoma Group (FNCLCC) grading system for soft tissue sarcomas was employed^5^. Surgical margins were classified as: R0, tumor was removed and cut off outside; R1, tumor was exposed to the sectioned surface. Figures showing mitotic activity in the largest cross-sectional slice of each tumor were identified; mitotic activity was expressed as the number of mitotic figures per 10 high-power fields (HPFs) (1 HPF = 0.24 mm^2^). The percentages of areas with necrosis, hemorrhage, fibrotic change, pleomorphism, and epithelial-like morphology in the largest cross-sectional slice of the tumor were calculated. Inflammation, histiocyte accumulation, cystic changes, rhabdoid cells, and giant cell components were also classified according to their inconspicuousness or prominence in the tumor.

**Figure 1:**
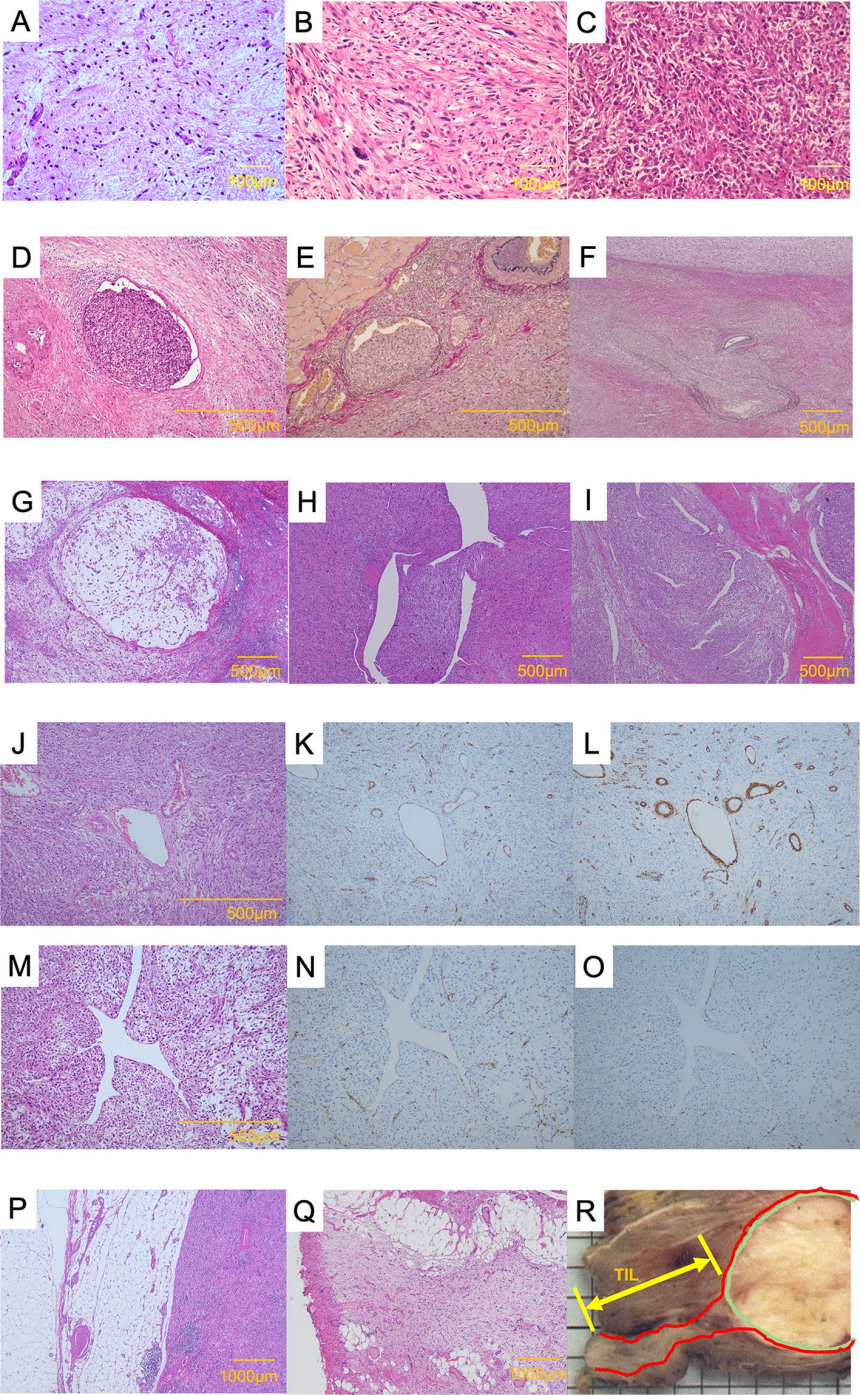
Cellularity classification. Hematoxylin and eosin (H&E) staining results showing (**A**) low (< 20%), (**B**) medium (20–50%), and (**C**) high (> 50%) cell contents. (**D**) Vascular invasion. (**E**) Elastica van Gieson (EVG) staining showing a small vascular invasion. Clefts are formed, and red blood cells are visible in the clefts. (**F**) Slightly larger vascular invasion with EVG staining, showing almost no cleft space. (**G**) Myxoid area in an undifferentiated sarcoma observed via H&E staining. (**H**) Sickle-shaped blood vessels observed via H&E staining. (**I**) Cluster of small sickle-shaped blood vessels observed via H&E staining. (**J**) Round-shaped blood vessels observed via H&E staining. (**K**) Anti-CD31 staining of the round-shaped blood vessel in panel j; the lumen is covered. (**L**) Anti-αSMA staining of the round-shaped blood vessel in panel j; positive cells are uninterrupted around the vessel. (**M**) Sickle-shaped blood vessels observed via H&E staining. (**N**) Anti-CD31 staining of vessels in panel m; the clefts are covered by the vascular endothelium, indicating a vascular lumen rather than an artifact. (**O**) Anti-αSMA staining of the sickle-shaped blood vessels in panel m, showing intermittent coverage by positive cells, mainly in the vascular branches. (**P**) INFa infiltration pattern with a clear border between the tumor and surrounding connective tissue observed via H&E staining. (**Q**) INFc infiltration pattern with tumor cells infiltrating into the surrounding connective tissue observed via H&E. (**R**) Differences in tail-like infiltration length (TIL) measurements between the macroscopic and microscopic tumor areas, showing gross (green line) and microscopic (red line) coverage extents.

Cellularity was classified as low (< 20%), medium (20–50%), and high (> 50%) using the classification system developed by Ulrich et al. (Fig. 1A,B,C)^6^. The area of the tumor section with the highest percentage cellularity was used as the cellularity value. Additionally, to evaluate vascular invasion, H&E staining and Elastica van Gieson (EVG) staining (Fig. 1D,E,F) were performed to identify tumor cells in vascular lumens, and while observing with a microscope, myxoid areas in the tumors were plotted on a macroscopic photograph, and areas were calculated using NIS-Elements D software (Nikon, Tokyo, Japan) (Fig. 1G).

#### Blood vessel evaluation

The sickle-shaped vascular pattern was defined as a fusiform dilated blood vessel with a bulge at its center and more than three branches or sharp ends (Fig. 1H,I). Further, among staghorn-shaped blood vessels with irregular branching, those with a pointed tip were defined as sickle-shaped, whereas those without a pointed tip were defined as round-shaped. The number of sickle-shaped blood vessels in the largest tumor slice for each sample was measured. Further, 10 tumor samples with conspicuous vasodilation were subjected to immunostaining for CD31 (PECAM-1; Leica Biosystems, Tokyo, Japan) and αSMA (1A4; Roche Diagnostics, Tokyo, Japan), and to confirm that these were vessels and not clefts due to artifacts, we checked coverage by the CD31-positive vascular endothelium. The percentages of αSMA-positive cells covering each of the 42 round and sickle-shaped blood vessels were calculated using an image analyzer (NIS-Elements D, Nikon Corporation, Tokyo, Japan) (Fig. 1J–O).

#### Infiltration pattern (INF) and tail-like infiltration length (TIL)

INF was defined based on microscopic findings as follows: INFa, expanding growth and distinct border with surrounding tissue; INFb, intermediate state between INFa and INFc; and INFc, infiltrating growth and indistinct border with surrounding tissue (Fig. 1P,Q). MFSs are characterized by a high frequency of local INF recurrence after surgery, which results in the presence of a characteristic tail-like pattern upon magnetic resonance imaging (MRI)^7^. Further, in this study, the difference between the coverage of microscopic and macroscopic tumor areas was defined as the tail-like infiltration length (TIL) (Fig. 1R).

#### RNA sequencing

A macroscopically vivid sample of surgically resected tumors with a minimum volume of 5 mm^3^ was first divided into approximate halves at the maximum split plane; one was embedded in Tissue-Plus O.C.T. (Thermo Fisher Scientific, Tokyo, Japan) for frozen sectioning, while the other was snap-frozen in liquid nitrogen and stored at -80 °C. A total of 24 tumor samples with more tumor cell-rich areas ≥ 30% based on the histological assessment of the mirror image surface of the tissue were subjected to RNA sequencing. Total RNA was isolated using the SV Total RNA Isolation System (F. Hoffmann-La Roche, Ltd., Basel, Switzerland), according to the manufacturer’s instructions. Thereafter, RNA purity was evaluated based on optical density ratios (A_260_/A_280_ and A_260_/A_230_) determined using a NanoPhotometer (Implen, Munich, Germany). Next, to assess RNA integrity, the RNA Nano 6000 Assay Kit and the Agilent Bioanalyzer 2100 system (Agilent Technologies, Santa Clara, CA, USA) were used. All the RNA samples thus obtained were subjected to quality inspection followed by library construction using the TruSeq Stranded mRNA Kit (Illumina, San Diego, CA, USA). This was followed by sequencing using an Illumina NovaSeq system at the Takara Bio laboratory (Shiga, Japan).

#### Transcriptome analysis

We used FastQ (https://www.bioinformatics.babraham.ac.uk/projects/fastqc/) to confirm read quality. Next, alignment to the human genome GRCh37 using STAR-2.5.2a (https://github.com/alexdobin/STAR/releases/tag/2.5.2a) and quality control analysis of the binary sequence alignment map (BAM) for each sample were performed using the Genomon2 RNA analysis pipeline (https://github.com/Genomon-Project). The mapped read count for each sample was then calculated using HTseq (https://htseq.readthedocs.io/en/release_0.9.1/) and normalized using the Relative Log Expression (RLE) method implemented in the DESeq2 Bioconductor R package (v1.26.0) (http://www.bioconductor.org/packages/release/bioc/vignettes/DESeq2/inst/doc/DESeq2.html). Further, RLE-normalized read count data were subjected to principal component analysis (PCA). Hierarchical clustering analysis of gene expression was also performed using 64 gene lists based on biological pathways from the KEGG, BioCarta, Gene Ontology, and Reactome databases. We also used 11 gene lists from the original study. Detailed information on the gene lists used is provided in Supplementary Table S1.

#### Statistical processing

The associations between clinicopathological findings and both metastasis-free survival and overall survival (OS) were analyzed using the Cox proportional hazards model, Kaplan–Meier method, *t*-test, Mann–Whitney U test, and the Pearson’s product moment correlation coefficient.

*P*-values < 0.05 (Cox proportional hazards model and Pearson’s product moment correlation analysis) were considered statistically significant. Further, the Kaplan–Meier method followed by the Bonferroni method for multiple comparisons was used, with *P* < 0.017 indicating statistical significance. All statistical analyses were performed using SPSS software v26 (SPSS Inc., Chicago, IL, USA).

### Ethics approval and consent to participate

This study was conducted with the approval of the Research Ethics Review Committee of Kanagawa Cancer Center (Approval number 2018 epidemiology-137) and was performed in accordance with the Declaration of Helsinki.

## Results

The patients were predominantly male, with a mean age of 70 years (Table 1). Their tumors, with a mean maximum diameter of 91 mm, were predominantly located in their thighs. Thirty-four tumors with R1 margins and 51 with R0 margins were excised. Local recurrence and distant metastases were observed in 22 and 29 of these cases, respectively. Metastasis to the lungs was the most common form of metastasis (19 cases), while fewer cases of metastases to soft tissue (five) and lymph nodes (three) were observed. Further, the cases were classified using the FNCLCC system as: G1, 13; G2, 37; and G3, 35^5^. The mean follow-up duration for these patients was 47 months. Detailed treatment histories were available for 57 cases. None of the patients received neoadjuvant therapy, and in some cases of positive resection margins, local radiation therapy was administered after surgery. Radiation therapy or systemic chemotherapy was also performed for patients who developed distant metastasis after surgery.

**Table 1:** Clinical and histological findings for 85 patients with myxofibrosarcoma/undifferentiated sarcoma.

Male/Female, n		62/23		Mitosis (10 HPFs)**		15 (7–32)	(0, 87)
Age, years*		70 ± 14	(18, 96)	Necrosis, %**		10 (0–30)	(0, 80)
Tumor size, mm*		91 ± 49	(18, 290)	Vascular invasion, n	V0	70	
Location, n	Thigh	35			V1	15	
	Lower leg	12		Sickle-shaped vascular**		10 (2–52)	(0, 1252)
	Upper arm	10			G1 (0–99), n	72	
	Forearm	7			G2 (100-), n	13	
	Shoulder	4		Myxoid area, %*		29 ± 37	(0.3, 100)
	Inguinal region	1		Myxoid area, n	0%	35	
	Back	4			0.1– 0.9%	4	
	Chest	4			1–49%	15	
	Buttocks	5			≧ 50%	31	
	Foot	1		INF a/b/c, n		43/24/18	
	Abdomen	2		Tail-like infiltration length (TIL), mm*		6 ± 10	(0, 45)
Dermis/Subcutaneous fatty tissue/Muscle		18/28/39		Cellularity, Low/Medium/High, n		15/34/36	
Margin, n	Positive (R1)	34		Hemorrhage area, %**		10 (0–20)	(0, 80)
	Negative (R0)	51		Fibrotic change, %**		10 (10–30)	(0, 60)
Local recurrence, n		22		Pleomorphic component, %**		40 (20–80)	(0, 100)
Time to local recurrence, month**		10 (5–21)	(2, 64)	Epithelioid like component, %**		0(0–20)	(0, 100)
Metastasis, n		29		Inflammatory change, inconspicuous/prominent, n	51/34		
Time to metastasis, month**		8 (4–17)	(0, 40)	Histiocytes accumulation, inconspicuous/prominent, n	75/10		
Metastatic organs, n	Lung	19		Cystic change, inconspicuous/prominent, n	67/18		
	Soft tissue	5		Rhabdoid cells component, inconspicuous/prominent, n	57/28		
	Lymph node	3		Giant cell component, inconspicuous/prominent, n	18/67		
	Brain	1		FNCLCC Grading	G1	13	
	Retroperitoneum	1			G2	37	
					G3	35	
				Follow-up period, months after surgery*		47 ± 34	(6, 164)

*Values are means ± SD (min, max).

**Values are median (interquartile range).

1 HPF = 0.2374 mm^2^.

**Figure 2:**
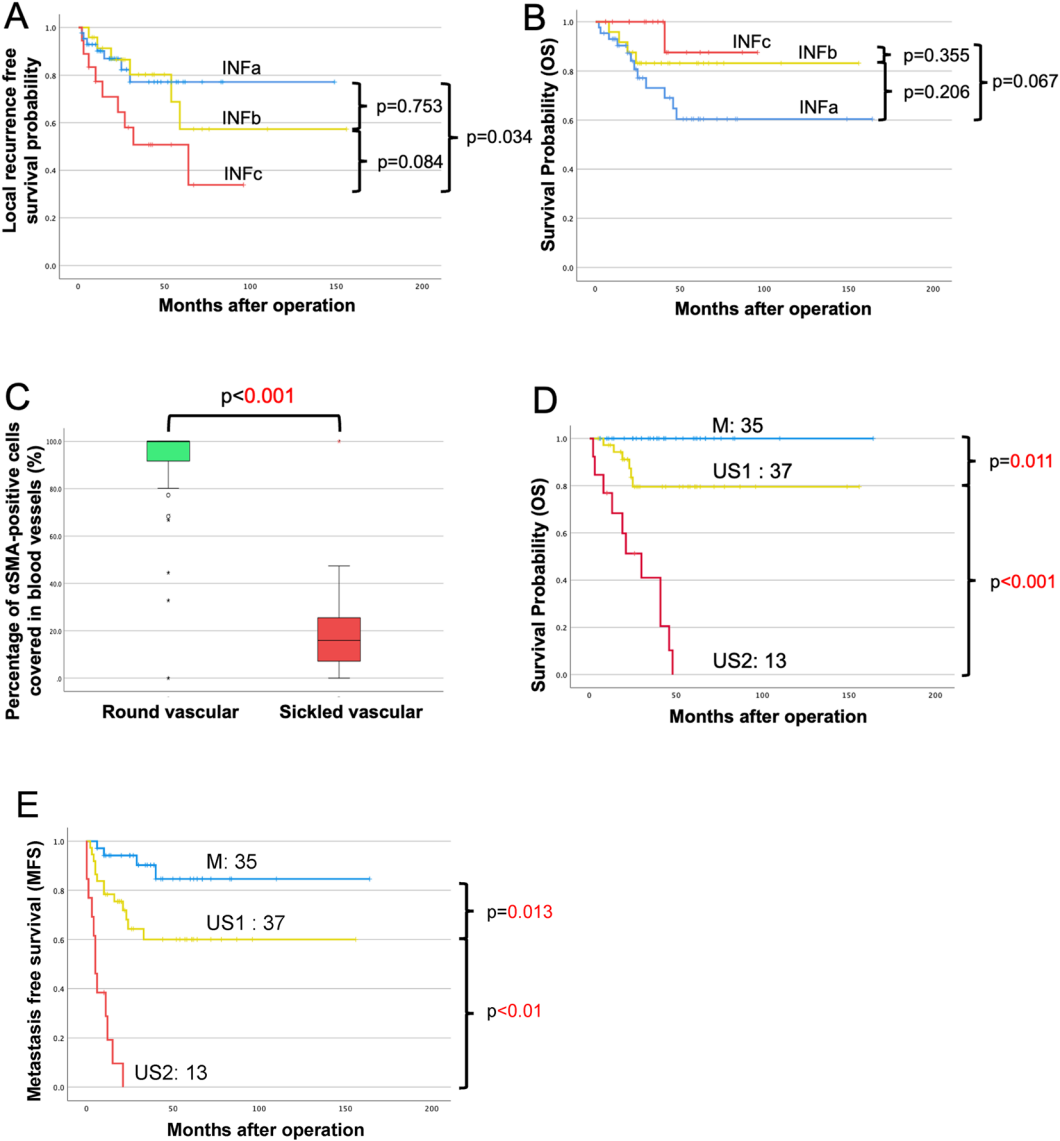
(**A**) Kaplan–Meier analysis of local recurrence and INF. Local recurrence tended to be more frequent in INFc cases than in INFa cases. (**B**) Kaplan–Meier analysis of overall survival and INF. The INFa group showed poorer prognoses than the INFc group. (**C**) Box-and whisker graph of αSMA-positive cells associated with 42 round and 42 sickle-shaped blood vessels. There was a significant difference in the percentage of αSMA coverage (*P* < 0.001, Pearson’s product moment correlation coefficient). (**D**) Survival of subgroups defined based on sickle-shaped blood vessel abundance. (**E**) Metastasis-free survival for the MFS, US1, and US2 cases.

There was a significant difference in local recurrence-free survival between the R0 and R1 surgical margin groups (*P* < 0.001, Cox proportional hazards model). Further, INFc cases tended to be more prone to local recurrence than INFa cases (Fig. 2A). In contrast, although the difference was not significant, INFc cases had relatively better prognoses than INFa cases with respect to OS (Fig. 2B). The mean TIL was 8 mm for INFb and 20 mm for INFc cases, and the maximum TIL was 45 mm (Table 2). For tumors with TIL < 10 mm, the proportion scored as R1 surgical margin was 26% (15 out of 57). In contrast, for patients showing TIL ≥ 10 mm, the proportion scored as R1 surgical margin was 68% (19 cases out of 28). Thus, there was a significant difference between the two groups (*P* < 0.001, *t*-test). In addition, there was a trend toward increased local recurrence in cases with TIL ≥ 10 mm compared with that in cases with TIL in the 1–9 mm range (*P* = 0.047, Kaplan–Meier method). We also observed that OS was not significantly different between cases with TIL in the 1–9 mm and TIL ≥ 10 mm ranges (*P* = 0.138, Kaplan–Meier method). The rate of occurrence of the R1 surgical margin was 16% and 83% in the INFa and INFc groups, respectively (*P* < 0.001, *t*-test). Further, the mean percentage of myxoid areas in INFa cases was 17%, compared to 41% and 38% for INFb and INFc cases, respectively. Our results also indicated that the proportions of myxoid areas observed in INFb and INFc cases tended to be higher than those in INFa cases (*P* = 0.001, Mann–Whitney U test).

**Table 2:** Tail-like infiltration length (TIL), resection margin assessment (R0, negative; R1, positive), and percentage of myxoid area for INFa, INFb, and INFc cases.

	n	TIL (mm)	(min, max)	R0 (%)	R1 (%)	Myxoid area (%)
INFa	43	0	(0, 0)	84	16	17 ± 30*
INFb	24	8 ± 6*	(0, 24)	50	50	41 ± 41*
INFc	18	20 ± 11*	(5, 45)	17	83	38 ± 40*

*TIL* Tail-like infiltration length.

*Values are means ± SD.

Based on univariate analyses, the characteristics that showed an association with OS included metastasis, mitotic activity, necrosis, sickle-shaped blood vessels, myxoid area proportion, epithelial-like structures, rhabdoid cells, and FNCLCC grade (Table 3). The hazard ratio for metastasis was as high as 325. Further, the parameters associated with distant metastasis included local recurrence, tumor diameter, mitotic activity, necrosis, vascular invasion, sickle-shaped blood vessels, and proportion of myxoid area. Vascular invasion, detected via H&E staining involving six cases and EVG staining involving nine cases, was not significantly associated with OS but was associated with metastasis-free survival (*P* < 0.001, Cox proportional hazards model).

**Table 3:** Associations between clinicopathological findings and overall survival.

	*HR (95% CI)*	*P*		*HR (95% CI)*	*P*
Gender (male, female)	0.838	0.757	Hemorrhage area (%)*	1.022	0.088
	(0.273–2.573)			(0.997–1.048)	
Age (years)	1.006	0.745	Fibrotic change (%)*	0.992	0.647
	(0.970–1.043)			(0.959–1.026)	
Local recurrence (positive, negative)	0.34	0.152	Pleomorphic component (%)*	1.008	0.291
	(0.078–1.490)			(0.993–1.023)	
Metastasis (positive, negative)	325.394	**0.027**	Epithelioid component (%)*	1.024	**0.003**
	(1.909–55,470.380)			(1.008–1.040)	
Tumor size (mm)	1.006	0.093	Inflammatory cells infiltration	0.629	0.384
	(0.999–1.013)			(0.222–1.786)	
Resection margin (positive, negative)	0.423	0.133	Histiocytes accumulation	1.426	0.578
	(0.138–1.300)			(0.409–4.966)	
Mitosis (10 HPF)*	1.027	**0.008**	Cystic change	0.929	0.699
	(1.007–1.047)			(0.638–1.351)	
Necrosis area (%)*	1.03	**0.002**	Rhabdoid cells component	3.046	**0.022**
	(1.011–1.049)			(1.171–7.920)	
Vascular invasion (positive, negative)	2.729	0.062	Giant cells component	0.376	0.055
	0.952–7.821			(0.138–1.022)	
Sickle-shaped blood vessels**	15.351	** < 0.001**	FNCLCC (G1, G2, G3)	6.351	**0.002**
	(5.585–42.2000)			(1.992–20.252)	
Myxoid area (%)*	0.966	**0.023**			
	(0.938–0.995)				

Statistically significant *P*-values are shown in bold.

*The HRs are for 1 mitosis or 1% increase.

**For analysis of sickle-shaped blood vessels, we dichotomized the cases based on the number of vessels in the maximum cut-surface of each tumor into those with 1–99 vessels and those with ≥ 100 vessels.

Patients with higher myxoid area proportions had more favorable prognoses (Table 3). The largest difference in the relationship between distant metastasis and OS was observed when the cases were divided into two groups using a 5% myxoid area cut-off (*P* ≤ 0.001, Kaplan–Meier method). The morphological characteristics of MFS, including percentage of myxoid area, invasive growth, cell density, and mitotic counts, were scored and classified into two groups (the so-called MFS group, 5 or 6 and so-called US group, 0–4) (Table 4). Specifically, the so-called MFS group showed a significantly better prognosis than the so-called US group, and none of the patients in the so-called MFS group died during the observation period *(P* < 0.001, HR 55.804, 95% CI 1.094–2846.131, Cox proportional hazards model).

**Table 4:** Histological scoring.

Myxofibrosarcoma grade (Histological parameter)	
Myxoid area	
Score 0	< 5% myxoid area
Score 2	5–49% myxoid area
Score 3	≧ 50% myxoid area
INF	
Score 0	INFa
Score 1	INFb or INFc
Cellularity	
Score 0	High cell content
Score 1	Low to medium cell content
Mitotic count	
Score 0	> 19 mitoses per 10 HPF
Score 1	0–19 mitoses per 10 HPF
Myxoid area + INF + Cellularity + Mitotic count Score	
So-called myxofibrosarcoma	Score 5, 6
So-called US	Score 0–4

High cell content: ≧ 50%.

Low to medium cell content: < 50%.

*HPF* high-power field; 1 HPF = 0.2374 mm^2^.

The means and standard deviations of the diameters of the 42 sickle-shaped and 42 round blood vessels were 64.5 ± 39.8 µm (min 19, max 185) and 65.1 ± 33.1 µm (min 13, max 174), respectively, and were not significantly different (*P* = 0.934, Pearson’s product moment correlation coefficient). The percentages of αSMA-positive cells covering the lumens of sickle-shaped and round blood vessels were statistically significant at 18.7 ± 17.7% (min 0, max 100) and 90.8 ± 20.5% (min 0, max 100), respectively (*P* < 0.001, Pearson’s product moment correlation coefficient) (Fig. 2C).

Further, the number of sickle-shaped blood vessels was associated with distant metastasis and OS, with a significant trend toward a poor prognosis (metastasis *P* < 0.001, Cox; OS *P* < 0.001, Cox) observed for patients with more than 100 such vessels in large maximal tumor sections. Of the 12 cases exhibiting vascular invasion, only four had more than 100 sickle-shaped blood vessels, and there was no correlation between vascular invasion and sickle-shaped blood vessels (*P* = 0.286, *t*-test). In MFS tumors, the mean number of sickle-shaped blood vessels was 18 ± 27, whereas in US tumors, the mean number of sickle-shaped blood vessels was not significantly different, at 77 ± 162 (*P* = 0.063, Pearson’s product moment correlation coefficient). None of the MFS tumors had > 100 sickle-shaped blood vessels. We also observed that in US tumors, distant metastasis was significantly increased in cases with a large number of sickle-shaped blood vessels, and OS was also poorer (metastasis *P* < 0.001, HR 6.986, 95% CI 2.896–16.855; OS *P* < 0.001, HR 7.476, 95% CI 2.719–20.554, Cox proportional hazards model). When we further divided the US group into the US1 and US2 subgroups (0–99 and > 100 sickle-shaped blood vessels, respectively), cases in the US2 group showed an association with significantly decreased OS and metastasis-free survival (Fig. 2D,E).

**Figure 3:**
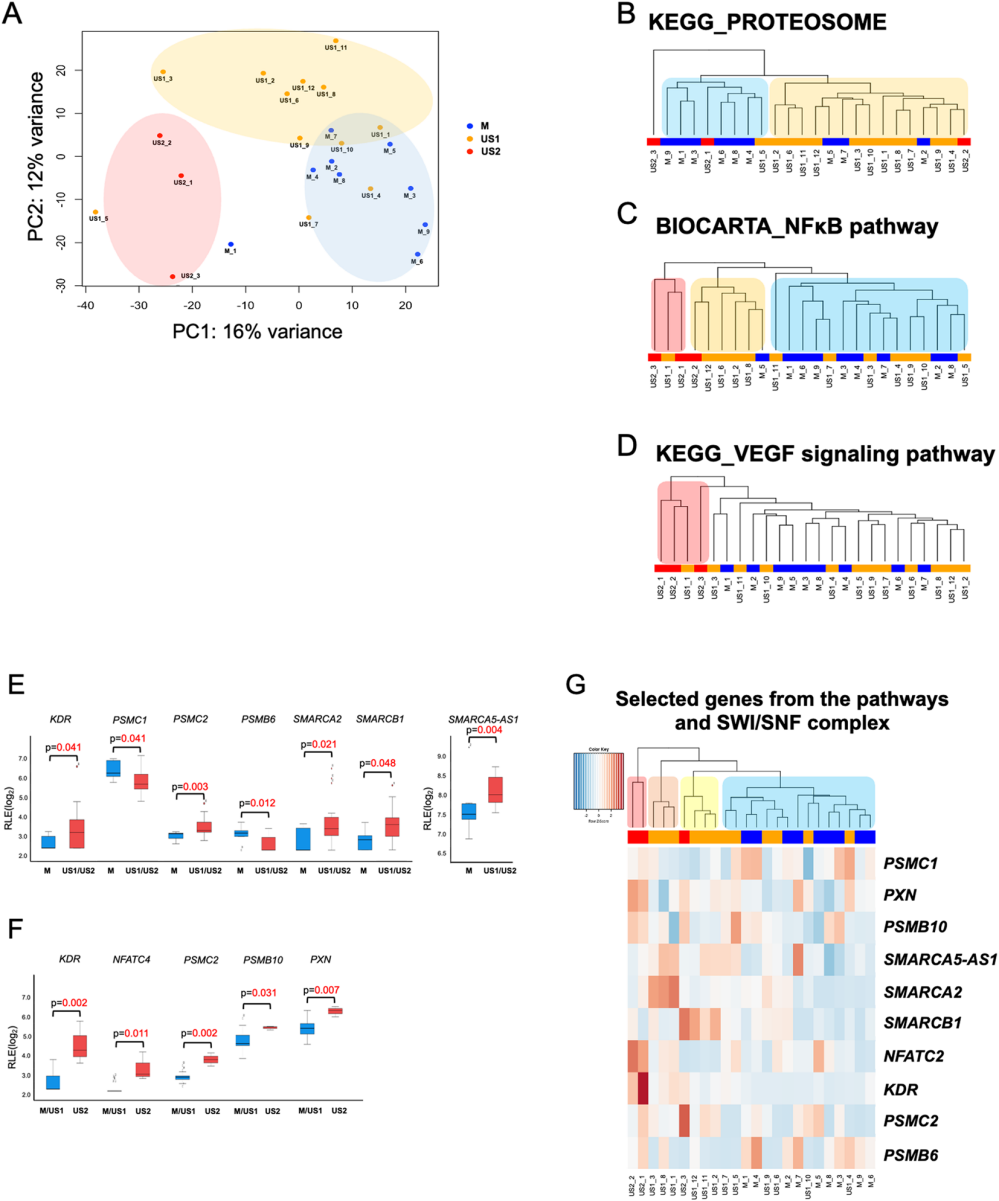
(**A**) PCA of gene expression profiles. Each case of so-called MFS (blue), US1 (orange), and US2 (red) is shown as a dot of a different color. Plots were clustered into the so-called MFS (blue frame), US1 (orange frame), and US2 (red frame) groups. (**B**), (**C**), and (**D**) Hierarchical clustering analysis for the KEGG_ PROTEASOME, BIOCARTA_NFκB, and KEGG_VEGF_signaling pathways, respectively. Cases of the so-called MFS, US1, and US2 clusters are shown in blue, yellow, and red boxes, respectively, alongside the case numbers. The identified clusters are shown using different colors (See text). Each complete result, with a heat map and the names of the individual gene in the pathway, is provided in Supplementary Figure S1. M, so-called MFS. (**E**) Box-and-whisker diagrams, analysis of genes in the PROTEASOME, *NFκB*, and *VEGF* signaling pathways and SWI/SNF complex-related sarcoma genes, which showed significantly different expression levels between the so-called MFS and US1/US2 (so-called US) groups, and (**F**) comparison between the so-called MFS/US1 and US2 (prominent sickle-shaped blood vessels) are shown. (**G**) Twelve genes with significant differences were used for the hierarchical clustering analysis. Cases of the so-called MFS, US1, and US2 groups are shown using blue, yellow, and red boxes, respectively, at the top of the heat map. Further, the clusters identified are shown using different colors (See text). The heat map shows the degree of change in the expression level of each gene according to the color range shown in the histogram (upper left). M, so-called MFS.

Among the 85 cases analyzed in this study, RNA sequencing data was available for 24 cases, including nine cases of MFS, 12 cases of US1, and three cases of US2. PCA of gene expression profiles was used to group the so-called MFS (blue frame), US1 (orange frame), and US2 (red frame) cases (Fig. 3A). Hierarchical clustering analysis of the 76 gene sets related to biological pathways showed that some pathways could be used to cluster the cases on the basis of the abundance of sickle-shaped blood vessels. Further, clustering analysis using the KEGG_PROTEASOME pathway set divided the 24 cases into two clusters and a US2 case. One cluster included the majority of MFS cases (6 out of 9), one US1 case, and one US2 case, whereas the other cluster consisted of 11 out of the 12 US1 cases, three MFS cases, and a single US2 case (Fig. 3B). Similarly, analysis using the BIOCARTA_NFκB pathway set clustered the 24 cases into three groups as follows: two US2 cases and one US1 case; one US2 case, four US1 cases, and one MFS case; and seven US1 and eight MFS cases (Fig. 3C). Clustering using the KEGG_VEGF_SIGNALING pathway gene set distinguished a group that included all three US2 cases and one US1 case from all the other groups (Fig. 3D). Next, we compared the expression levels of each gene in the three pathways (Supplementary Table S2) between the MFS/US groups. The expression levels of *PSMC1* and *PSMB6* in the PROTEASOME pathway and *KDR* in the VEGF signaling pathway were significantly higher; the expression level of another proteasomal gene, *PSMC2,* was significantly lower in MFS tumors than in US1/US2 tumors (Fig. 3E). Further, when we grouped MFS and US1 together, we identified lower *PSMC2* expression level as one of its characteristic features; the proteasomal gene *PSMB10* showed the same behavior (Fig. 3F). Furthermore, the expression levels of *NFATC4* and *PXN* of the VEGF signaling pathway, in addition to the expression level of *KDR,* were significantly higher in US2 cells (Fig. 3F). However, the expression levels of *VEGF* and *NFKB1* were not significantly different between MFS and US1/US2 tumors or between MFS/US1 and US2 tumors (data not shown).

The SWItch Sucrose Non-Fermentable (SWI/SNF) complex orchestrates gene expression by remodeling chromatin compaction, and its components are often perturbed in soft tissue neoplasms^8^. Given that gene expression profiles based on biological pathways could distinguish the MFS or MFS/US1 from other tumor types, we next measured the expression levels of SWI/SNF complex genes, such as *SS18*, *SMARCA1*, *SAMRCA2*, *SMARCA4*, *SMARCA5*, *SMARCA5-AS1*, *SMARCAD1*, *SMARCAL1*, *SMARCB1*, *SMARCC1*, *SMARCC2*, *SMARCD1*, *SMARCD2*, *SMARCD3*, and *SMARCE1*, which are often perturbed in sarcomas. Thus, we observed significantly higher *SMARCB1*, *SMARCA2*, and *SMARCA5-AS1* expression levels in the US (US1/US2) group (Fig. 3E).

Finally, we selected genes that showed significantly different expression levels between the newly defined MFS/US groups based on the three abovementioned biological pathways and also examined the SWI/SNF complex genes for hierarchical clustering analysis. Thus, we clustered the 24 cases into four groups, namely, US2, US1 with prominent *SMARCA2* expression, US1 with prominent *SMARCB1* expression, and MFS plus a few US1 cases (Fig. 3G).

## Discussion

MFS, characterized by a myxoid stroma, and US, which can also exhibit a myxoid stroma, are tumors of the same lineage. However, there is an obvious difference in prognosis between them, and clear criteria by which they can be distinguished in terms of myxoid stroma amount have not yet been reported. In this study, we observed that MFS/US prognoses could be clearly stratified using several histologically appreciable parameters, including myxoid stroma amount, tumor infiltration pattern, and number of sickle-shaped blood vessels. In particular, blood vessel morphological classification was correlated not only with prognosis but also with gene expression profiles, suggesting differences in the biological backgrounds of the tumors. The examination of the degree of cellular pleomorphism did not show any relationship with OS; thus, realizing an objective evaluation in this regard was challenging. We also examined tumor depth but did not observe any relationship with OS. Further, we investigated the presence of curvilinear blood vessels, which are considered specific to MFS histologically. However, defining and evaluating the degree of curvilinear blood vessels was also challenging; thus, it could not be evaluated as an objective parameter. Based on the results of our examination of several histological parameters, the above parameters could be evaluated relatively objectively based on the histological features of MFS/US. Further, they were also considered to be related to OS.

In the INFb and INFc groups, distant metastases were less frequent, and OS tended to be better. Further, both groups had higher myxoid area percentages than the INFa group. Although MRI-based tail-like findings have been reported, their relationship with TIL as defined in this study remains unclear. The local tumor recurrence rate was significantly higher for patients in the INFc group and with TIL ≥ 10 mm. Further, the percentage of positive resection margins increased significantly when TIL was ≥ 10 mm, and the percentage of completely resected cases for the INFc group was only 17%, suggesting that the invasive growth pattern of the tumor affected local recurrence. The absence of tumor exposure at the resection margin showed an association with local recurrence (*P* = 0.011, Cox proportional hazards model); therefore, if we can predict INF and TIL preoperatively, it may contribute to a more complete resection, hence reducing local recurrence. INF and TIL involve subjective judgments. Thus, it is necessary to evaluate inter-observer judgments, test–retest reliability, and establish some easy-to-use criteria for mitigating the discrepancies associated with subjective judgments. It may also be necessary to introduce these parameters into routine diagnosis.

Attempts have been made to distinguish between MFS and US based on myxoid area proportion, and the use of a myxoid area of 10% as a cut-off value has been reports recommended^9^. In this study, we began by collecting cases of US and MFS that were previously classified under the same disease group (MFH) and analyzed the associated factors indicating poor prognosis. We noted that cut-off values of 0.1, 1, 5, 10, 20, 30, 40, 50, 60, 70, 80, 90, and 100% were all associated with OS (Supplementary Table S3). Thus, there is no doubt that myxoid area affects MFS/US prognosis. Our results further showed that a 5% cut-off yielded the most significant difference in prognosis. However, objective histological measurement of myxoid area is difficult; hence, distinguishing between MFS and US based only on myxoid area remains challenging. In this study, we scored multiple morphological features to yield two groups, MFS and US, that correlated well with prognosis.

Although the significance of sarcoma pathology has not yet been established, in addition to the morphological features, the presence of vascular invasion evaluated via both H&E and EVG staining showed a significant correlation with distant metastasis, which in turn showed a correlation with the prognosis of patients with MFS/US. In nine of the 15 (60%) invasion-positive cases, EVG staining was necessary to identify vascular invasion, showing its utility for predicting distant metastases.

**Figure 4:**
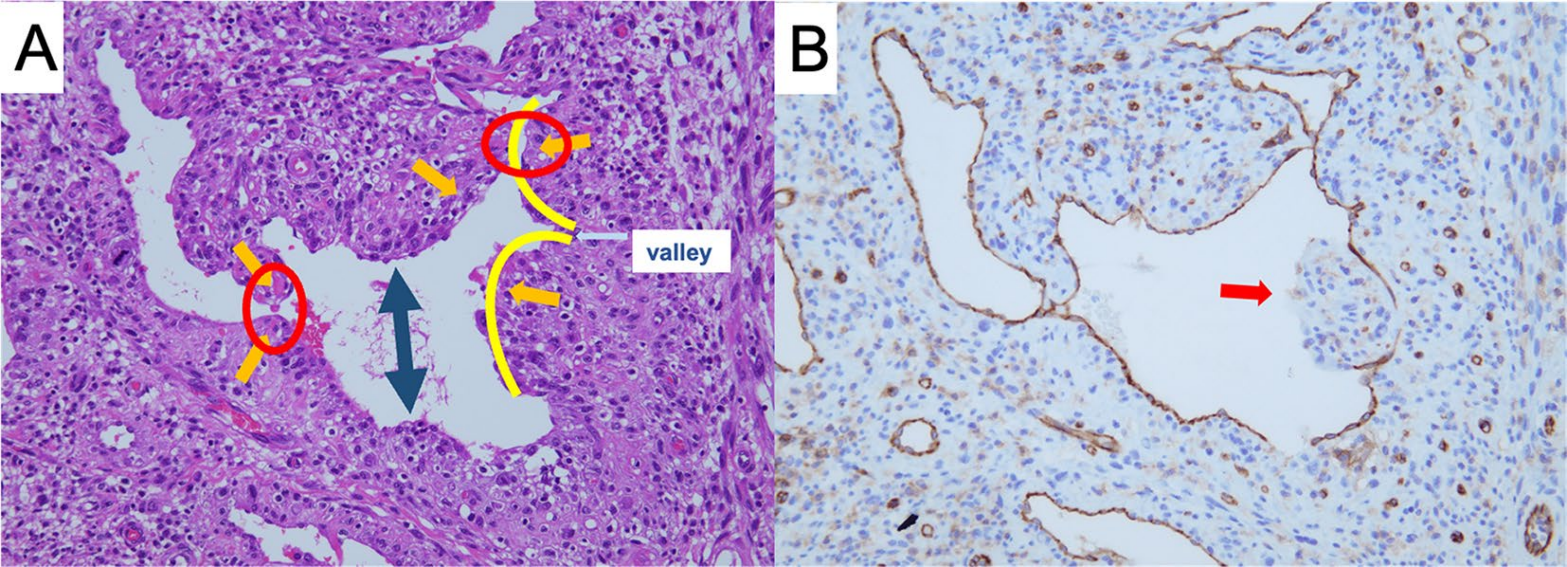
(**A**) Sickle-shaped blood vessels as observed after H&E staining. Possibly, due to weak vascular support structures, irregular stenosis (red circle) caused by surrounding tumor cell growth (orange arrow) and pressure on the intervening vessels (blue arrow) can be observed. The tumor may be growing as multiple mountainous protrusions within the dilated vessels, causing valleys between them, steep bifurcations, and sharp angles at the tips. (**B**) Sickle-shaped blood vessels in CD31-stained cells. CD31-positive endothelial cell covering is shed in some of the intravascular protrusions (red arrow).

Staghorn-shaped blood vessels are occasionally observed in multiple types of sarcomas, including solitary fibrous tumors and synovial sarcomas. Sickle-shaped blood vessels that are even more finely defined than staghorn-shaped blood vessels, which are dilated and show bifurcations (Fig. 4A), are significantly associated with OS, and may be a powerful prognostic factor that can be assessed via H&E staining. While there was a significant tendency for more frequent distant metastasis in cases with prominent sickle-shaped blood vessels, no clear relationship with vascular invasion using EVG staining was observed. Reportedly, even though αSMA is not a specific marker of pericytes, it is considered one of the markers of mature pericytes^10^. In this study, sickle-shaped blood vessels had significantly fewer αSMA-positive cells covering them, compared with round-shaped vascular vessels. This may be due to the absence or immaturity of pericytes lining the blood vessels. Pericytes, which are specialized cells located on the abluminal surface of capillary blood vessels, perform key functions in vascular homeostasis^11^. They also exhibit several characteristics consistent with muscle cell activity and express contractile smooth muscle actin^12^; therefore, they are commonly referred to as muscle cells^12^. Glioblastomas and mammary carcinomas exhibit a more dramatic reduction in pericyte density, compared with normal tissues^12^. The precise causes of this abnormal pericyte density remain unknown but may include imbalanced endothelial cell/pericyte signaling circuits and/or a limited pool of recruitable pericytes^13^. In this study, the percentage of αSMA coverage in the sickle-shaped blood vessels tended to be lower than that in normal round-shaped vasculature, suggesting that roundness decreased due to a lack of smooth muscle differentiation. Pericytes are also involved in vascular permeability, and immature vessels that are not lined with pericytes are prone to cancer cell infiltration, which is reportedly associated with hematogenous distant metastasis and a poor prognosis in colorectal cancer^14^. The same may be true for sarcomas. Sickle-shaped blood vessels are characterized by dilation, steep branching, and sharp angles at their tips. One possible reason for these characteristic morphologies is that the vessels are only partially pressurized and are dilated due to irregular stenosis caused by the growth of tumor cells into immature vessels; this weakens the support structures of vascular walls. Thus, tumor cells protrude in a mountainous fashion against blood vessels, creating valleys between them. This may produce steep bifurcations and sharp angles at the tips. Immunostaining with a relatively new block showed inconsistent findings with no CD31-positive vascular endothelial covering in some of the sickle-shaped blood vessels, consistent with the observed protrusions and suggestive of vascular invasion (Fig. 4B). However, to test this hypothesis, it would be necessary to assess, in detail, the presence of pericytes around sickle-shaped like blood vessels as well as their maturity and the expression status of proteins and genes related to the vascular endothelium, vascular pericytes, and growth factors of angiogenesis using a larger number of cases.

In this study, we first subclassified the MFS/US sarcoma group into the MFS and US groups. Taking the abundance of sickle-shaped blood vessels into consideration, we further subclassified US as showing a high abundance of in sickle-shaped blood vessels (US2) or their absence (US1). This grouping of MFS as US1 and US2 showed a good correlation with patient prognosis, with the US2 group showing the shortest OS. Our transcriptome analyses confirmed the significance of our morphological classification. PCA was further used to cluster the cases into three groups. One case of MFS (M-1) was close to the area of US2. Interestingly, this case M-1 showed a high abundance of sickle-shaped blood vessels (n = 82), suggesting that it may have some characteristics of US2.

Gene amplification and the elevated expression of the proteasome pathway gene *SKP2*, which encodes a subunit of ubiquitin ligase SCFs, are reportedly associated with a poor prognosis in patients with MFS^15^. Although the expression of *SKP2* was not significantly different in our subgroups of MFS/US tumors, several other proteasome pathway genes were differentially expressed. Particularly, the expression levels of other proteasome pathway genes, including *PSMC1*, *PSMB6*, *PSMC2*, and *PSMB10* seemed to be differentially expressed in MFS/US sarcomas. Similarly, the expression levels of SWI/SNF-related genes, such as *SMARCB1*, *SMARCA2,* and *SMARCA5-AS1* also seemed to be differentially expressed in MFS/US sarcomas. The biological or clinical meanings of these differential expression levels remain uncertain. Thus, further studies involving a larger number of cases are necessary.

High VEGF levels induce the development of tortuous, leaky, and immature blood vessels^17,18^; anti-VEGF therapy has been shown to normalize these characteristics, partly by increasing coverage by pericytes^19^. Although we observed no significant difference in the expression level of *VEGF*, the expression level of *KDR*, which encodes one of the two VEGF receptors, was upregulated in US tumors, particularly in US2 tumors. Moreover, the expression levels of the other two VEGF signaling pathway-related genes, *PXN*, encoding paxillin^20^, and *NFATC4*, encoding a DNA-binding transcription complex originally characterized in activated T cells^21^, were upregulated in US2 tumors. Recently, *NFATC4* was characterized as a transcription factor that regulates the expression of neuritin, an angiogenic factor^20^ that signals via the insulin receptor^21^. This suggests that multiple angiogenic stimuli are involved in the establishment of sickle-shaped blood vessels.

Myxofibrosarcoma grade and sickle-shaped blood vessels have the potential for indicating prognosis based on histological findings only. Prognostication is considered important for follow-up and treatment strategies. However, the limitation of this strategy is that the further validation of myxofibrosarcoma grading and sickle-shaped blood vessels based on other cohorts is often necessary. MFS has a characteristic histology and tends to show a better prognosis than US, although both tumors are considered to be of the same spectrum. Histological scoring showed good performance in the stratification of the prognosis of patients with MFS/US. We also observed consistency between gene expression profiles and histology-based stratifications. Further, given that distant metastasis is an important factor for indicating prognosis in MFS/US tumors, the abundance of sickle-shaped blood vessels, which we precisely defined in this study, can be a useful predictor in this regard. Increased expression of angiogenesis-promoting genes has also been observed in sickle-shaped blood vessel-rich tumors. As only a limited number of tumors were available for RNA sequencing in this study, further analyses involving a larger number of tumor cases with abundant sickle-shaped blood vessels may help to elucidate the pathogenesis of the malignant phenotypes of MFS/US tumors and also highlight targets for developing therapeutics for this disease.

## Supplementary Information

Supplementary Information 1.

Supplementary Information 2.

Supplementary Information 3.

## Data Availability

The datasets generated and/or analyzed during this study are not publicly available because they are saved on private servers but may be available from the corresponding author upon reasonable request.
